# Panomics to decode virulence and fitness in Gram-negative bacteria

**DOI:** 10.3389/fcimb.2022.1061596

**Published:** 2022-11-21

**Authors:** Anuradha Singh, Bindu Ambaru, Viraj Bandsode, Niyaz Ahmed

**Affiliations:** Pathogen Biology Laboratory, Department of Biotechnology and Bioinformatics, University of Hyderabad, Hyderabad, India

**Keywords:** Gram-negative bacteria, antimicrobial resistance, multi-omics, integrative data analytics, host-pathogen interactions

## Introduction

Over the millennia, Gram-negative bacteria (GNB) have evolved to become one of the leading causes of fatalities across the globe. These bacterial species range from colonizers of the mammalian gut to pathogenic clones, often implicated in foodborne outbreaks and hospital-associated infections (HAIs) (Janda and Abbott, 2021; Ruegsegger et al., 2022). Eradication of these pathogens is further challenged by the emergence of multidrug-resistant (MDR) phenotypes and lack of novel drugs in the discovery pipeline (Laxminarayan et al., 2016). Recent estimates show that bacterial antimicrobial resistance (AMR) was responsible for 4.95 million recorded death cases in 2019, ranking third among all other global disease burdens (GBD) (Murray et al., 2022). It is noteworthy that six of the twelve pathogens mentioned by the WHO (https://www.who.int/initiatives/glass/glass-routine-data-surveillance; Veeraraghavan and Walia, 2019) were GNB, highlighting the need for a deeper understanding of the likely molecular mechanisms behind the propensity and fitness of these pathogens including their interactions with hosts.

With leaping whole genome sequencing (WGS) data, understanding of the molecular and genetic mechanisms underlying the evolution of bacterial pathogens from commensals to pathogens has considerably improved over the past two decades. Acquisition of genetic variation through horizontal gene transfer (HGT) and genome reduction are two major events responsible for bacterial evolution and colonization in diverse host and environmental contexts (Ahmed et al., 2008). Amongst GNBs such as *Escherichia coli*, *Salmonella* spp., *Klebsiella pneumoniae*, *Pseudomonas aeruginosa*, and *Acinetobacter baumannii*, HGT-mediated acquisition of mobile genetic elements (MGEs) such as plasmids, phages and, genomic islands (GIs) remains the predominant mechanism of genome evolution (Hawken and Snitkin, 2019). These MGEs harbor genes encoding virulence factors and AMR to avert host-defense mechanisms and environmental vulnerabilities thus providing a survival advantage. The evolution of *E. coli* strains into diverse sequence types (STs) such as ST73, ST131, and ST95 provides substantial evidence regarding the role of HGT in genomic fine-tuning and pathogenicity (Forde et al., 2019; Shaik et al., 2022). High-throughput computational studies from our previous work (Suresh et al., 2021) demonstrated HGT-mediated dissemination of polyketide synthase (*pks*) island across different STs and serotypes of *E. coli*, which is often implicated in colorectal malignancies. Genome reduction is another major evolutionary force observed among Mycobacteria. Compared to other Gram-negative pathogens and host generalist species of *Salmonella*, pseudogenization-mediated metabolic fine-tuning in the immediate host niche appears to be a predominant mechanism of the genome evolution in *Salmonella* Typhi strains (Baddam et al., 2014).

Given that complex adaptive processes exhibited by the bacteria operate in a network of interactions spanning several molecular layers, the response of an entire cellular system to a given perturbation cannot be adequately captured from a single layer. Deep and accurate knowledge to develop a holistic molecular perspective of a biological system requires not just one but several omics analyses. Heterogeneous datasets derived from different omics platforms such as genomics, transcriptomics, proteomics, metabolomics, metagenomics, meta-transcriptomics, meta-proteomics, and meta-metabolomics may complement each other and offer an attractive approach to understand the organisms as well as their interactions with corresponding hosts. Hence, the aim of this review is to discuss and build a narrative on multi-omics/panomics research on Gram-negative priority pathogens and to emphasize upon the need to harness integrated omics analyses to comprehend and control life-threatening infections.

## Multi-omics analyses to decode intricate biological processes of Gram-negative bacteria

The current state of art elucidates several omics methodologies and interdisciplinary approaches which have surpassed the traditional ones. This section highlights some of the studies that have used two or more omics layers to shed light on the GNB’s dynamic biological processes. We discuss them taking examples from some of the known priority pathogens.

### Escherichia coli

Humans are susceptible to a wide range of intestinal and extraintestinal diseases and infections caused by *E. coli*. The enteric *E. coli* are divided into different pathotypes such as enteropathogenic, enterotoxigenic, enteroinvasive, enterohemorrhagic, enteroaggregative, and diffusely adherent based on their virulence traits (Kaper et al., 2004; Ahmed et al., 2008). Antibiotic-resistant *E. coli* is commonly employed as a model organism in structural and functional investigations to comprehend the physiology and gene expression of MDR bacteria. By using an integrated multi-omics approach that includes the genomic, transcriptomic, and proteomic data of enterohemorrhagic *E. coli* (EHEC) EDL933, Cho and colleagues investigated the interactions between host mucin and pathogen proteins, providing a valuable resource for the creation of Lectin-Glycan Interaction Network (LGI) of *E. coli* (Cho et al., 2020). Extracting critical phenotypic alterations responsible for drug resistances was made possible by the integration of transcriptomics and genomics data (Suzuki et al., 2014). Comparative genomics, transcriptomics, and functional characterization (Hazen et al., 2017) demonstrated that hybrid-pathogenic strains of *E. coli* are capable of expressing the virulence genes from various pathovars. Some of the transcriptomics and fluxomics studies (Fong et al., 2006) enabled new insights into the evolutionary dynamics of *E. coli* by demonstrating the flexibility of the metabolic network to countervail genetic perturbations and also emphasized the advantage of combining multiple omics datasets to differentiate between causal and noncausal mechanistic changes. Another important work (Piazza et al., 2018) predicted a network of interactions and binding sites in *E. coli* using a metabolomics and proteomics approach, thus allowing the discovery of novel enzyme-substrate interactions. Genome-scale metabolic models (GEMs) of *E. coli* B and *E. coli* K12 constructed by integrating the comparative analyses of genomes, transcriptomes, proteomes, and phenomes provided the basis for differentiating the two strains. Similar studies providing insights into cellular physiology and metabolism could be relevant for engineering microorganisms for bioprocess applications as well as towards understanding the virulence mechanisms of various pathogens (Yoon et al., 2012).

### *Salmonella* spp.

*Salmonella*’s rising antibacterial resistance and the lack of novel antimicrobials on the horizon are being addressed *via* multi-omics studies. Proteomics, metabolomics, glycomics, and metagenomics were used in a multi-omics ‘systems’ approach (Deatherage Kaiser et al., 2013) to investigate the molecular interactions between *Salmonella enterica* serovar Typhimurium (*S.* Typhimurium), the murine host, and the microbiome during intestinal infection with *S.* Typhimurium. Proteogenomics was employed recently (Karash et al., 2017) to identify the potential genes and proteins that play a role in *S.* Typhimurium's resistance to H_2_O_2_, thus deepening the current understanding of *S.* Typhimurium's survival mechanisms in macrophages. Another research (Crouse et al., 2020) focused on integrating WGS techniques into food safety practices could establish links between virulence and genetic diversity in *Salmonella*. They have also presented a novel approach for risk assessment of particular strains as well as for improved monitoring and source tracking during outbreaks. By utilizing metabolomics and transcriptomics, it has been possible to understand that both glycolysis and lipid metabolism were regulated by SlyA in *Salmonella* (Tian et al., 2021). Another study based on high throughput analyses (Hossain et al., 2017) harnessed the advantage of genomics, gene expression analysis, proteomics, metabolic pathways, and subcellular localization to discover 52 distinct essential proteins in the target proteome of the *S. enterica* that could be used as novel targets to develop newer drugs. Utilizing metabolomics and transcriptomics, it was possible to assess adaptation of *S.* Typhimurium to essential oils (thyme and cinnamon) and to study the induced resistance as well as the underlying adaptive mechanisms (Chen et al., 2022). Recently, a promising therapeutic target that activates immune response against the extremely drug-resistant (XDR) strain called *S.* Typhi H58 has been successfully identified using a comprehensive strategy of computational reverse vaccination along with subtractive genomics (Khan et al., 2022).

### Klebsiella pneumoniae

It is challenging to treat infections caused due to MDR and highly virulent *K. pneumoniae* strains, highlighting the urgent need to discover novel and effective therapeutics against this pathogen. This was addressed (Ramos et al., 2018) by integrating various multi-omics data like genomics, transcriptomics, metabolomics, and protein structure information to delineate 29 proteins with preferential properties for therapeutic development against *Klebsiella*. This work also provided insights into *K. pneumoniae* metabolism under various host-imitating circumstances. Recently, a gene and metabolite-centric network-based method (Cesur et al., 2019) identified potential therapeutic targets for *K. pneumoniae*, MGH 78578. A thorough assessment of the identification of pharmacological targets and their implications in the therapeutic management of *Klebsiella* infections was presented (Ali et al., 2022) using a multi-omics perspective.

### Pseudomonas aeruginosa

AMR nosocomial pathogen *P. aeruginosa* is currently posing unwavering and increasing threats to humans. Grady and colleagues (Grady et al., 2017) integrated the results from studies including RNA-Seq, proteomics, ribosome footprinting, and small molecule LC-MS, to compare the gene expression of *P. aeruginosa*. Collectively, their findings unleash the mechanisms underlying the bacteria’s ability to grow and survive on n-alkanes. Integrated analysis of transcriptomics and metabolomics revealed that polymyxin therapy significantly altered lipid, lipopolysaccharide, and peptidoglycan biosynthesis as well as central carbon metabolism and oxidative stress (Han et al., 2019). This study also demonstrated the systems-level dynamics of polymyxin-induced cellular responses, highlighting the need for combination therapy to reduce resistance to the last-resort therapeutic option, polymyxins. Further, it was possible (Filho et al., 2021) to integrate transcriptome data with genome-scale metabolic networks of *P. aeruginosa* to identify potential therapeutic targets. Rashid and colleagues (Rashid et al., 2017) used a comprehensive subtractive genome and proteome computational framework in their investigation to predict potential *P. aeruginosa* vaccine candidates. Recently, a multi-omics based investigation (Molina-Mora and García, 2021) incorporating genomics, phenomics, comparative genomics, transcriptomics, and proteomics provided new insights about molecular determinants of antibiotic resistance in a MDR strain of *P. aeruginosa* (PaeAG1).

### Acinetobacter baumannii

*A. baumannii’s* exceptional propensity to quickly acquire resistance determinants to a wide range of antibiotics has made it a significant global cause of HAIs. Understanding the pathophysiology and evolution of AMR can help us fight illnesses caused by *A. baumannii*. Clinical isolates of *A. baumannii* have been reported to be resistant to triclosan (Chen et al., 2009). A multi-omics investigation employing WGS, transcriptomics, and proteomics was carried out to better understand the global alterations in protein expression in the triclosan-resistant mutant strain, AB042 to understand the mechanisms of resistance (Fernando et al., 2017). According to their findings, *A. baumannii* reacts to triclosan by changing the expression of genes related to amino acid and fatty acid metabolism, and AMR. The colistin resistance mechanism in MDR-ZJ06, an MDR clinical strain of *A. baumannii*, was elucidated (Hua et al., 2017) by combining genomics, transcriptomics, and proteomics. The loss of bacterial lipopolysaccharide (LPS) caused by ISAba1 insertion in lpxC was identified in their investigation as the resistance mechanism of the colistin-resistant strain. Through the integration of various data sources, including the co-expression, operon organization, and associated protein structural data of genes in *A. baumannii* (Xie et al., 2020), a co-functional network was built with potential AMR and virulence related features.

Public data sharing is an essential component of research to fight against pathogens. The growing accessibility of microbial omics data combined with heterogeneous metadata is revolutionizing the study of infectious diseases and numerous resources are being created to organize such enormous amounts of data. The prominent microbiological databases that incorporate multi-omics and multi-(meta) omics datasets as well as the specialized databases that focus on a particular GNB are listed in **Table 1**. In addition, it also includes technologies/resources available for data integration. A more comprehensive list can be found in the database-focused annual edition of Nucleic Acids Research (Rigden and Fernández, 2022).

**Table 1:** Prominent microbiological databases and resources available for data integration.

Database	Omics data types	Functionality	Organisms	URL	Reference
Bacterial and Viral Bioinformatics Resource Centre (BV-BRC)	Genomics Transcriptomics Proteomics Metabolomics Metagenomics	Provides access to a variety of data for the National institute of Allergy and Infectious Diseases (NIAID’s) priority pathogens.	NIAID category A to C/emerging/reemerging pathogens	https://www.bv-brc.org/	(Davis et al., 2020)
Omics Discovery Index (Omicsdi)	Genomics Transcriptomics Proteomics Metabolomics Metagenomics	An open-source platform that provides access, discovery and dissemination of omics data sets. Currently, 11 different repositories,hosted on 4 continents are included in this database.	Various microorganisms	http://www.omicsdi.org	(Perez-Riverol et al., 2017)
National Centre for Biotechnology Information (NCBI)	Genomics Transcriptomics Proteomics Metabolomics Metagenomics Metatranscriptomics Metaproteomics	NCBI establishes standards for data deposition and exchange for the scientific and medical sectors, as well as access to a variety of databases and tools. NCBI provides access to a variety of databases and software, promotes standards for data deposition and exchange for the scientific and medical communities	Various microorganisms	https://www.ncbi.nlm.nih.gov/	(Schuler et al., 1996)
European Molecular Biology Laboratory - European Bioinformatics Institute (EMBL-EBI) European Life-Science infrastructure (ELIXIR)	Genomics Transcriptomics Proteomics Metabolomics Metagenomics Metatranscriptomics Metaproteomics	Provides bioinformatics resources for promoting research, and disseminates cutting-edge technologies to the academic community and industry.	Various microorganisms	https://www.ebi.ac.uk/ https://elixir-europe.org/	(Kanz et al., 2005)
DNA Databank of Japan (DDBJ)	Genomics Transcriptomics Proteomics Metabolomics Metagenomics Metatranscriptomics Metaproteomics	Provides services for depositing and retrieving sequencing data, software tools for analyzing biological data	Various microorganisms	https://www.ddbj.nig.ac.jp/index-e.html	(Tateno et al., 2002)
China National GenBank (CNGB)	Genomics Transcriptomics Proteomics Metabolomics Metagenomics Metatranscriptomics Metaproteomics	A unified platform created for the research community’s application services and sharing of biological big data.	Various microorganisms	https://db.cngb.org/	(Guo et al., 2020)
Joint Genomic Institute Integrated Microbial genomes (JGI-IMG)	Genomics Transcriptomics Proteomics Metabolomics Metagenomics Metatranscriptomics Metaproteomics	Supports the annotation, analysis and distribution of microbial genome and microbiome datasets.	Various microorganisms	https://img.jgi.doe.gov/	(Markowitz et al., 2014)
Metagenomic Rapid Annotations using Subsystems Technology (MG-RAST)	Genomics Transcriptomics Metagenomics Metatranscriptomics	A Metagenomic analysis server for microbial communities	Various microorganisms	https://www.mg-rast.org/	(Keegan et al., 2016)
BioCYC	Genomics Transcriptomics Proteomics Metabolomics Regulatory networks	The tools for omics data analysis, comparative genomes, and comparative pathway analysis are all provided by BioCyc. It has distinct home pages for the different organisms like EcoCyc.org for *E. coli*; Salmonella.biocyc.org for *Salmonella* spp	Various microorganisms	https://biocyc.org/	(Karp et al., 2019; Keseler et al., 2021)
Ecomics	Transcriptomics Proteomics Metabolomics Fluxomics Phenomics	Multi-omics compendium for *E. coli*	*E. coli*	http://prokaryomics.com/	(Kim et al., 2016)
SalmoNet	Protein-protein interactions, transcriptional regulatory interactions and enzyme-enzyme interactions	A molecular interaction database providing network resource containing regulatory, metabolic and protein-protein interactions	*Salmonella*	http://salmonet.org/	(Métris et al., 2017)
SYSTOMONAS	Genomics Transcriptomics Proteomics Metabolome Gene regulatory network	A source for *Pseudomonas* systems biology analysis	*Pseudomonas*	http://www.systomonas.de.	(Choi et al., 2007)
Klebnet	Genomics	A platform for genomic surveillance with analytics, specifically designed for the complex of *K. pneumoniae* species	*Klebsiella*	https://klebnet.org/	(Lee et al., 2019)
ABviresDB	Resistance or virulence features as well as their co-functional interactions.	ABviresDB will be useful in revealing the mechanisms of bacterial resistance and virulence and for the network study of bacterial infection	*Acinetobacter*	https://acba.shinyapps.io/ABviresDB/	(Xie et al., 2020)
**Resources for omics data integration**					
Omics integrator	Genomics Transcriptomics Proteomics Metabolomics Pathway analysis Visualization	Holistic analysis of different types of omics datasets	Various Microorganims	http://fraenkel-nsf.csbi.mit.edu/omicsintegrator/	(Tuncbag et al., 2016)
Paintomics 3.0 (web based)	Genomics Proteomics Metabolomics Pathway analysis	A web application for visual representation of integrated view of several omic datasets	Various Microorganisms	https://www.paintomics.org/	(García-Alcalde et al., 2011)
integrOmics (R package)	Genomics Proteomics Metabolomics Microbiome	Two ‘omics’ variables that are measured on the same samples are effectively integrated by integrOmics.	Various Microorganisms	http://CRAN.R-project.org/	(Lê Cao et al., 2009)
mixOmics (R package)	Genomics Transcriptomics Proteomics Metabolomics Visualization	Focused on data exploration, dimension reduction, and visualisation with a particular emphasis on multivariate analysis of biological data sets.	Various Microorganisms	http://www.bioconductor.org/packages/release/bioc/html/mixOmics.html	(Rohart et al., 2017)
ProteoClade (Python)	Genomics Proteomics Metaproteomics	Associate taxonomic studies of several species with proteomic data	Various Microorganisms	http://github.com/HeldLab/ProteoClade	(Mooradian et al., 2020)
Metaboanalyst 5.0	Proteomics Metabolomics Pathway analysis Visulaization	Analysis, interpretation, and integration of metabolomics data with other omics data	Various Microorganisms	https://www.metaboanalyst.ca/	(Pang et al., 2022)
Qiime2	Metagenomics Metatranscriptomics Metaproteomics Metabolimics	Open-source microbiome analysis tool that transforms unprocessed sequence data into understandable visualisations and statistical findings	Various Microorganisms	https://qiime2.org/	(Caporaso et al., 2010)

## Holo-omics approach for deciphering the host-pathogen interactions

Extremely intricate interactions exist between microorganisms and host cells, and these interactions are not always uniform or linear in nature. Pathogens alter the primary metabolic processes in themselves as well as in the host cell based on the nutrient sources prevailing in the infected host niche. This necessitates a comprehensive strategy that can take into account the various data types using the same inference framework so as to broaden the scope of investigations on microbes and hosts. ‘Holo-omics’ investigations incorporate information from many omic levels in the host and microbial domains (Nyholm et al., 2020). Expanding the scope of biological interpretation and examining biological pathways in greater detail are made possible by the ability to integrate many meta-omics levels, like meta-genomes, meta-transcriptomes, meta-proteomes, and meta-metabolomes. Epigenomic and exposomic profiling is made possible by similar technologies, and this can help to further disentangle the biochemical interactions between host-microbiota and environment and their impact on host phenotypes (Kumar et al., 2014; Rogler and Vavricka, 2015). GEMs offer a better comprehension of how intracellular infections make use of the host’s existing milieu. The host-cell nutritional environment and gene expression data from *S.* Typhimurium grown inside macrophage cell lines were used to study *Salmonella* metabolism during infection (Raghunathan et al., 2009). *Salmonella*’s metabolic changes proceeding from the early stages of infection until chronic infection was predicted by simulations of the GEM (iRR1083) (Raghunathan et al., 2009). Their data reveal occurrence of a minimal set of metabolic pathways that is necessary for *Salmonella* to successfully replicate inside the host cell. Additionally, this model provides a framework for the identification of networked metabolic pathways, incorporation of high-throughput data to produce hypotheses regarding metabolism during infection, and the logical development of new antibiotics. Another work (Ding et al., 2016) used *in silico* metabolic modeling to predict the crucial genes of enterobacterial human pathogens (*E. coli* and *Salmonella* strains) in different host habitats, including the human bloodstream, urinary tract, and macrophage for understanding the pathogen’s survival and infection mechanisms. It is possible to explore condition-specific pathogenicity by mapping multi-omics data to GEMs. Although the technology to produce huge amounts of data for use in a holo-omics environment is currently available, the data integration methods to uncover and detect host-microbe interactions are still limited, thus opening new avenues towards applied research.

## Way forward: Integrative data analytics

Data integration approaches broadly fall into two distinct categories depending upon the assumption as to whether the biological variation is unidirectional or multidirectional i.e, multi-staged analysis and meta-dimensional analysis (Ritchie et al., 2015; Jendoubi, 2021).

A multi-staged analysis refers to the integration of data in a hierarchical or stepwise manner wherein only two different data types are combined at once to investigate the relationship between them. In contrast, meta-dimensional analysis refers to simultaneous integration of multiple variables from different data types (Ritchie et al., 2015). Though meta-dimensional analysis is statistically more robust as compared to multi-staged analysis, it also increases the dimensionality of the data while combining many data types, making it more complex to interpret. The choice of data integration approach primarily depends on the aim of the study along with other factors such as sampling, omics platforms, and quality of the data (Graw et al., 2021). Recently, such a multi-dimensional approach has been used for drug target prioritization in MDR *K. pneumoniae* (Ramos et al., 2018).

Further, meta-dimensional analyses could be categorized into three different methods depending upon the stage of data integration i.e, concatenation-based (early integration), transformation-based (intermediate integration), and model-based (late integration) (Ritchie et al., 2015). In concatenation-based methods, data gathered across various omics platforms could be combined to create a joint matrix that serves as an input dataset for machine learning algorithms. This approach has been used to study stress response in *E. coli* wherein a combined dataset of transcriptomics and metabolomics was used as an input for machine learning algorithms. K-means clustering and canonical clustering analysis (CCA) were used to understand the coordinated changes in transcripts and metabolites under different stress conditions (Jozefczuk et al., 2010).

In transformation-based methods, data are first transformed into intermediate forms such as graph and kernel matrix followed by integration into a combined matrix and data analysis. As the data are transformed into intermediate form, this particular strategy of integration preserves the characteristics of each unique data type. Different machine learning frameworks have been developed to learn from transformed datasets. DeepDRK is one such deep learning model which involves kernel-based integration of multi-omics data to predict drug response of cancer cell lines (Wang et al., 2021).

In model-based approach, individual omics datasets are first used as training datasets to build respective models and finally, multiple models are integrated to mine biological processes. MOMA (Multi-Omics Model and Analytics) (Kim et al., 2016) is one such platform wherein model-based data integration was used to study the cellular states of *E. coli* under unexplored conditions.

Although multi-omics data integration techniques have lately gained popularity in a number of scientific domains, this area of study is still in its infancy in case of bacterial species. Given the exponential increase in multi-omics data, integrated analytics may prove to be one of the most effective methods to comprehend both the basic as well as stress physiology of bacteria. This strategy can assist biomedical researchers in discovering strain-specific biomarkers thereby elucidating cellular mechanisms of pathogenesis and developing novel therapeutic approaches.

## Open challenges and future directions

**Figure 1:**
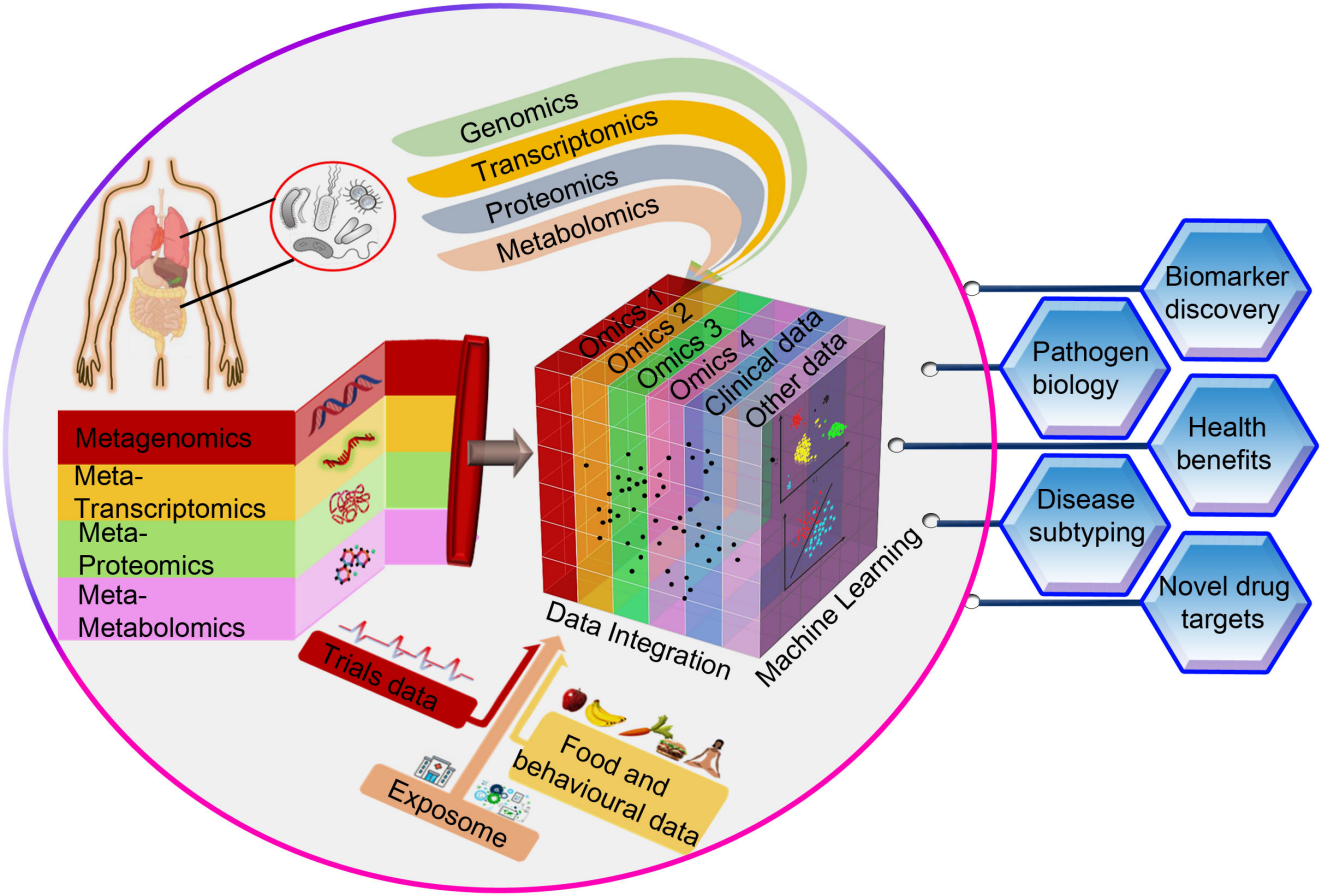
A conceptual framework for integrating omics data (pathogen-specific data and community-level data) with non-omics datasets such as clinical metadata through machine learning techniques. This approach is very likely to advance our understanding of infectious diseases and accelerate the identification of novel diagnostic or therapeutic targets that can improve human health.

The advantage of panomics data integration to get a holistic understanding of biological processes and infection mechanisms has its own inherent challenges. Multi-omics analyses present additional obstacles such as methods to be used for integration, clustering, visualization, and functional characterization on top of the difficulties that single-omics analyses entail (Pinu et al., 2019). For instance, researchers may encounter difficulties with data harmonization (data scaling, data normalization, and data transformation methods pertaining to individual omics datasets) prior to combining two or more omics datasets. Furthermore, the computational resources and storage space needs can be prohibitive for a given study due to dimensionality limits when integrating huge datasets. Our ability to integrate pathogen-specific omics data, community-level omics data and the non-omics datasets such as clinical metadata will improve the understanding of infectious diseases and hasten the discovery of new diagnostic or therapeutic targets (**Figure 1**). Despite inevitable practical, financial, and computational challenges, the incorporation of various multi-omics data types from both the microbe and the host sides could revolutionize the understanding of infections caused by AMR bacteria. Given this, in-depth analyses of the disease coordinates, both at the levels of pathogens and hosts, would be beneficial in devising personalized treatments.

## Author contributions

AS and BA contributed equally to the writing of the review. VB prepared table. NA conceptualized the opinionated content, and contributed to editing and finalizing the manuscript. All authors contributed to the article and approved the submitted version.

## Acknowledgments

We thank the Indian Council of Medical Research (ICMR) for a grant awarded to NA (AMR/257/2021/ECD-II). We also acknowledge the University of Hyderabad for providing facilities and institutional support (Institution of Eminence -PDF; financial support to UoH-IoE by MHRD [F11/9/2019-U3A]). AS acknowledges the Junior Research fellowship from the Department of Biotechnology, Government of India and the Prime Minister's Research Fellowship (PMRF) from the Indian Government.

## Conflict of interest

The authors declare that the research was conducted in the absence of any commercial or financial relationships that could be construed as a potential conflict of interest.

## Publisher’s note

All claims expressed in this article are solely those of the authors and do not necessarily represent those of their affiliated organizations, or those of the publisher, the editors and the reviewers. Any product that may be evaluated in this article, or claim that may be made by its manufacturer, is not guaranteed or endorsed by the publisher.

## Abbreviations

GNB, Gram-negative bacteria; HAIs, Hospital-Associated infections; MDR, Multidrug-Resistant; AMR, Antimicrobial Resistance; GBD, Global Disease Burdens; WHO, World Health Organization; WGS, Whole Genome Sequencing; HGT, Horizontal Gene Transfer; MGEs, Mobile Genetic Elements; GIs, Genomic Islands; ST, Sequence Type; *pks*, Polyketide Synthase; EHEC, Enterohemorrhagic *E. coli;* LGI, Lectin-Glycan Interaction network; GEMs, Genome-Scale Metabolic models; XDR, Extremely Drug-Resistant; LPS, Lipopolysaccharide; CCA, Canonical Clustering Analysis; MOMA, Multi-Omics Model and Analytics.
